# Testing the efforts model of simultaneous interpreting: An ERP study

**DOI:** 10.1371/journal.pone.0206129

**Published:** 2018-10-24

**Authors:** Roman Koshkin, Yury Shtyrov, Andriy Myachykov, Alex Ossadtchi

**Affiliations:** 1 NRU Higher School of Economics, Moscow, Russia; 2 Center of Functionally Integrative Neuroscience, Department of Clinical Medicine, Aarhus University, Aarhus, Denmark; 3 Department of Psychology, Northumbria University, Newcastle-upon-Tyne, United Kingdom; Universitat Zurich, SWITZERLAND

## Abstract

We utilized the event-related potential (ERP) technique to study neural activity associated with different levels of working memory (WM) load during simultaneous interpretation (SI) of continuous prose. The amplitude of N1 and P1 components elicited by task-irrelevant tone probes was significantly modulated as a function of WM load but not the direction of interpretation. Furthermore, the latency of the P1 increased significantly with WM load. The WM load effect on N1 latency, however, did not reach significance. Larger negativity under lower WM loads suggests that more attention is available to process the source message, providing the first electrophysiological evidence in support of the Efforts Model of SI. Relationships between the direction of interpretation and median WM load are also discussed.

## Introduction

Unlike in monolingual communication, in simultaneous interpreting (SI) a message in one language is perceived and processed almost concurrently with the production of an equivalent message in another language. To be able to accomplish this feat, besides high proficiency in both the source and target languages, the interpreter must possess a set of specialized skills, including exceptional language switching abilities [1], large working memory (WM) span [2], ability to manipulate WM content and understand incoming discourse while producing a rendering of an earlier portion of the source message in the target language. By its nature, SI is externally paced [3,4], indicating the need for cognitive resource management and coping strategies [5–10].

In SI, an interpreter usually begins interpreting before the speaker has finished a sentence. The speaker, however, does not normally wait to move on to the next utterance, regardless of whether the interpreter has completed the translation of the previous chunk [5,11]. Moreover, it may not always be possible or convenient to maintain sequential linearity of the target message relative to the source. For example, interpreters often reverse the order of lists. In some language combinations, e.g. German/English, syntactic constraints force one to wait for the final verb in the German source to construct the target sentence in English [12]. Finally, the interpreter may choose to defer translating a word until a good enough equivalent comes to mind, hoping to be able to work it into the target message later. The resulting source-target lag—also referred to as *décalage* or *ear-voice-span (EVS)* in the interpretation studies literature—between the source and the target messages highlights the critical role of WM in the SI pipeline. WM represents a mental space within which to perform the transformations needed for a coherent and accurate target message to emerge.

Under normal circumstances, when the source message is relatively easy to understand and target equivalents are quickly and automatically retrieved from long-term memory (LTM), the interpreter maintains a comfortable décalage, accurately rendering the source message with almost no omissions. But when confronted with a long-winded, dense or obscure passage, the interpreter may be forced out of the comfort zone and temporarily increase the lag to accommodate the need for more time to process it. The lag is similar to debt in that beyond a certain point it becomes difficult to handle. In extreme cases, when the interpreter gets too far behind to speaker, performance quality may be compromised: parts of the source message may get severely distorted or go missing from the translation altogether. This may happen when the interpreter has shifted much of his/her attention away from the currently articulated source chunk to finish processing the previous one stored in WM, in order to catch up with the speaker. In sum, large lags are most likely caused by processing difficulties.

On the other hand, when the source message is overall relatively difficult to follow (e.g. when the message is not in the interpreter’s mother tongue), the interpreter may need to allocate extra effort towards understanding. This can be done by shortening the décalage, effectively limiting the amount of information to be processed in working memory. Such a strategy may result in a more literal translation that is likely to be syntactically and grammatically deficient.

In our opinion, the above considerations are best captured by Gile’s Efforts Model [13] which conceptualizes SI in terms of three groups or mental operations, or ‘efforts’: listening, production and memory. Since these efforts are mostly *non-automatic* and *concurrent*, they critically depend on and compete for the limited pool of attentional resources. A major implication of the model is that increased processing demands in one of the efforts can only be met at the expense of another. In fact, several studies involving dual-task situations indirectly support this view suggesting that transient performance decreases in one task occur due to the engagement of attention in another task (e.g. [14]).

To our knowledge, only one study has attempted to test the Efforts Model of SI experimentally [15]. But as its author himself admitted, “it cannot be said to have led to [its] systematic testing or validation” and also suggested that “precise quantitative measurement” would help to make it more useful. To address this concern (at least partially), in the present paper we used the ERP technique to test one particular prediction of the Efforts Model, namely that increased processing demands on the ‘memory effort’ means less processing capacity available to the ‘listening effort’ (that involves active processing of the input heard). In other words, a higher WM load would create a deficit of attention to the auditory stream. Whereas this hypothesis may seem quite intuitive, to our knowledge, it has never been tested experimentally in a naturalistic setting requiring the participants to interpret continuous prose overtly. Electrophysiological evidence supporting it would suggest that interpreters’ brains gate part of the auditory input to be able to properly process the information backlog and reduce the associated processing pressure. Here and throughout we refer to the ‘memory’ and ‘listening’ efforts as defined by Gile [13].

We exploited the previous findings that N1 and even P1 [16] amplitude evoked by task-irrelevant probes embedded in a speech stream is modulated by selective attention in what is called ‘processing negativity' observed as early as 50–150 ms from the stimulus onset [17]. Specifically, the ERP waveform appears shifted towards negative values when the listener attends the target audio (e.g. [18–23]). Moreover, a more recent EEG study [24] showed that in a multitasking situation—and SI is an extreme case of multitasking [25]—increased WM load decreases attention to the targets. Therefore, the parameters of these early auditory ERP components can be used as a suitable and temporally precise index of interpreters’ attention to the spoken *source* message.

Our assumption that WM overload reduces attention to the auditory stream, which in turn modulates the ERP waveform, aligns well with the evidence that both WM and attention may utilize a common pool of neural resources [26]. As demonstrated by fMRI studies, attention and WM are subserved by overlapping brain areas [27–33].

While according to some studies–notably, not involving the Russian-English language pair used here–interpreters may prefer to interpret from L2 into L1 (e.g. [34,35] but see [36]), in our survey conducted prior to this study, out of 32 professional simultaneous interpreters (English-Russian/Russian-English, L1 Russian speakers), 29 reported that, all else being equal, interpreting from L2 into L1 was much more difficult than in the opposite direction. Furthermore, the interpreters we surveyed also said the most difficult part for them was to *understand* the source message in L2—and understanding is part of the ‘listening effort’ according to Gile [13,15,37]. Based on these observations and the prediction of the Efforts Model we expected that this subjective difficulty would result in a significant difference in median WM loads between L1→L2 and L2→L1 directions of interpretation.

Being aware that the early P1 and N1 ERP components produced by task-irrelevant tone probes are not known to be sensitive to the language of the speech that these probes are embedded into, we nevertheless wished to check whether the direction of interpretation would have an *indirect* effect on the amplitude of these early ERP components. The rationale for this was as follows. Given that bilinguals demonstrate superior memory performance for L1 words [38,39], we assumed that for L1 Russian/L2 English interpreters English→Russian interpreting involves a greater memory effort than Russian→English interpreting. If the Efforts Model is correct in predicting that larger memory effort means less attention to the current auditory input, we can hypothesize that increasing the lag (and by assumption WM load) by N words in the L2→L1 direction of interpreting should decrease P1/N1 negativity by a *larger* amount than when the lag is increased by the same number of words in L1→L2 interpreting.

Importantly, achieving the goals of the study required defining a method of estimating WM load. The most obvious and straightforward approach to do it would be to simply assume that WM load is proportional to the number of words that an interpreter lags behind the source message at any given time. In the time intervening between the moment of hearing a word and finishing its translation overtly, the interpreter has to store it in WM at least until it is further processed as appropriate to the situational context. If we assume that the cognitive effort it takes to process the most frequent function words—especially articles and prepositions—is negligible, we can exclude them from WM load estimation. However, this approach would be an oversimplification. First, it is predicated on the assumption that each word strains WM capacity equally. More frequent words must have stronger mental representations and a larger network of associations than relatively rare ones. In what is known as the *word frequency effect* [40–42] more frequent words are processed faster. Therefore, they must be translated more easily and cleared from working memory relatively quickly to make room for processing the next chunk of the source message. Conversely, words that are less familiar take more time and effort to process, which delays the retrieval of equivalents from LTM and increases WM load. Second, such an approach does not take into account that words in the source message are never chunked together such that they are stored in WM as a single mental object. Third, it assumes that WM load is reduced at the moment when a word has been translated overtly. This is not always the case because interpreters continue to post-process their own translation, in a phenomenon known as self-monitoring [37,43–46]. This means that after a word (or, more generally, a chunk) is translated overtly, it is not cleared from WM immediately, but at a later time, when the interpreter is satisfied with his or her translation. An opposite situation may happen—and this is the fourth reason—when the interpreter can use informational redundancy of the source message to predict an idea *before* it is fully uttered by the speaker [5]. For example, if the source message has multiple references to the United Nations Organization, it is easy to finish the translation before the offset of the phrase *the United … [Nations Organization]*, in other words get ahead of the speaker. The above considerations mean that WM load estimates will be inherently noisy. To mitigate this error of measurement, we sought to maximize the sample and explored several alternative methods of WM load estimation, which we describe below.

## Methods

### Participants

Nine males (aged 25–47, *M* = 36.9, *SD* = 6.757) participated in the study. All were qualified interpreters holding a university degree in translation and interpreting, L1 Russian/L2 English speakers, with an average of 10.65 (*SD* = 6.675) years of professional SI experience. None of them reported having any hearing-related problems, substance abuse or a neurological disorder. The experiment was approved by the Institutional Review Board of the National Research University Higher School of Economics, Moscow. All the participants signed an informed consent form.

### Procedure and materials

Each of the participants was asked to interpret 8 speeches originally delivered at the 6849th United Nations Security Council Meeting by representatives of eight countries: Chile, Colombia, Costa Rica, Honduras, Peru, Uruguay (in Spanish) and France and Morocco (in French). Since we were interested in the Russian-English language combination, we used 4 Russian translations and 4 English translation (see S1 and S2 Appendices for details) available on the official UN website at http://www.un.org. The topics discussed in the speeches centered on the rule of law and were unlikely to elicit a strong emotional response. We deliberately chose speeches originally delivered in a language other than the participants’ L1 or L2. This was necessary to avoid a potential bias due to some subtle properties (e.g. idiosyncratic syntax) that may have been present in the translated, but not in the original texts, and vice versa.

We expected that UN terminology would be familiar enough to the participants so that they could deliver quality translation with no preparation. To control for a potentially confounding effect of varying delivery speed, we had the written Russian and English translations read at a slow constant rate by a bilingual speaker (female) highly proficient in both Russian and English. After the recording we used Audacity (http://audacityteam.org), open audio editing software, to edit the recordings to a constant delivery rate of 105 words per minute (wpm). Thus we eliminated possible confounds that could have been introduced due to individual speaker’s voice features such as rate, pitch, timbre, loudness, prosody and accent. The total playback time was about 54 minutes (excluding periods of rest between the speeches).

The source speech recordings and task-irrelevant probe stimuli (440-Hz 52-ms pure sine wave tones including a rise and fall period of 4 ms) were played by a custom script running under PsychoPy [47]. The specific choice of 52 ms was a tradeoff between the need to maximize the amplitude of the expected ERPs (by longer probes) and minimize the masking of the source audio by too long probes. The probes were delivered throughout the entire performance of the interpreting task simultaneously to both the right and left ear with a jittered inter-stimulus interval (ISI) of 450–750 ms (*M* = 600 ms). These parameters were selected empirically to maximize the number of probes per second of experimental time while minimizing the effect of diminished ERP amplitude with shorter ISIs [21].

To control for order effects, the speeches were delivered to the participants in a pseudo-random fashion according to a Latin square design such that for every participant the texts’ order was different.

The speech recordings were played through earbud headphones (Sennheiser MX 170, 32Ω, Germany). Before the experiment, the participants did two 30-second practice runs, in which they were asked to adjust the volume to a comfortable level and interpret an excerpt from a speech delivered at the same UNSC session, but not included in the experimental material.

Participants were seated comfortably in a reclining chair in an electrically and acoustically shielded room. To reduce oculo-motor and muscle artifacts, they were instructed to sit still, relax, minimize eye movements and articulate their translation as quietly as possible. The translation was recorded using a Boya BY-M1 capacitor microphone for subsequent offline transcription and timecoding.

### Working memory load estimation

The number of content words in the source-target lag provides a measure of instantaneous WM load. However, a potentially more precise estimate could be achieved by scaling every content word by its min-max normalized log-transformed frequency, $\hat{f}$, obtained from a representative language corpus. Min-max normalization maps a word’s log-transformed absolute frequency in the corpus, *f*, to a value in the range between 0 and 1, while log-transformation helps ensure a better fit for the linear model describing the relationship between a word’s rank and frequency in the corpus. For example, the WM load associated with the word ‘peace’, ${\hat{f}}_{"\mathrm{peace}"}$, after controlling for its frequency in the corpus is given by:

$${\hat{f}}_{"peace"}=\frac{\log(f_{"peace"})-\log(F_{\min})}{\log(F_{\max})-\log(F_{\min})}$$ (1)

where *F*_max_ and *F*_min_ are the absolute frequencies, respectively, of the most and the least frequent word in a given corpus, and *f*_*”peace”*_ is the absolute frequency of the word ‘peace’ in the corpus. Estimating WM load this way seems appropriate since word frequency is a major factor determining word recognition [48]. More frequent words elicit quicker responses than less frequent ones [49,50]. In fact, Whaley [51] argued that word frequency was “by far the most powerful predictor” in a lexical decision task. For source texts we chose the Russian ruTenTen (14.5 billion words) and the British National Corpus (112.2 million words) accessed through Sketchengine interface (https://www.sketchengine.co.uk/). Before looking up a word’s frequency in the corpus, we used the NLTK package [52] to lemmatize each word.

The methods described above are predominantly based on the number of content words. However, they can be further improved by scaling each word according to its syllabic length, a potentially important factor influencing the speed of processing. This approach makes particular sense since the phonological loop—an essential component of auditory WM [53,54]—has a capacity of about 2 s unless the information stored in it is constantly rehearsed and/or processed.

**Fig 1:**
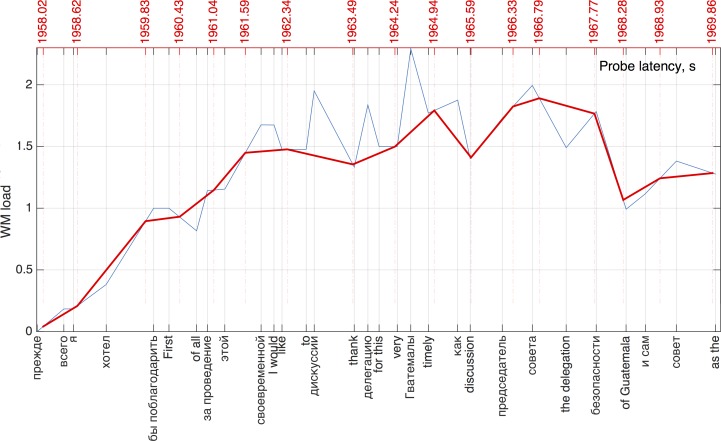
A slice of a time-coded transcript showing WM load dynamics over time. The blue line represents WM load at word offsets. The red line captures linearly interpolated values of WM load at probe onsets. Red numbers along the top axis show probe onset times. WM load was estimated using the CL method.

In summary, we used three different methods to estimate WM load, specifically in the number of (a) content words (CW); (b) content words weighted by their frequency (CL), (c) all the words (both function and content) weighted by their respective syllabic length (SYL). In this study function words included articles, prepositions, auxiliary verbs and the conjunction “and” for English; prepositions and the conjunction “и” for Russian. Changes in WM load were assumed to occur at the time of word offset. Since the probes were delivered at random intervals not corresponding to word offsets, we used linear interpolation to estimate WM load at probe onsets (Fig 1). Given the high probing density (on average every 0.6 s), it may be assumed that the probes coincided with roughly the same ideas in the source text across participants and across directions.

### Transcript time coding and WM load estimation

**Fig 2:**
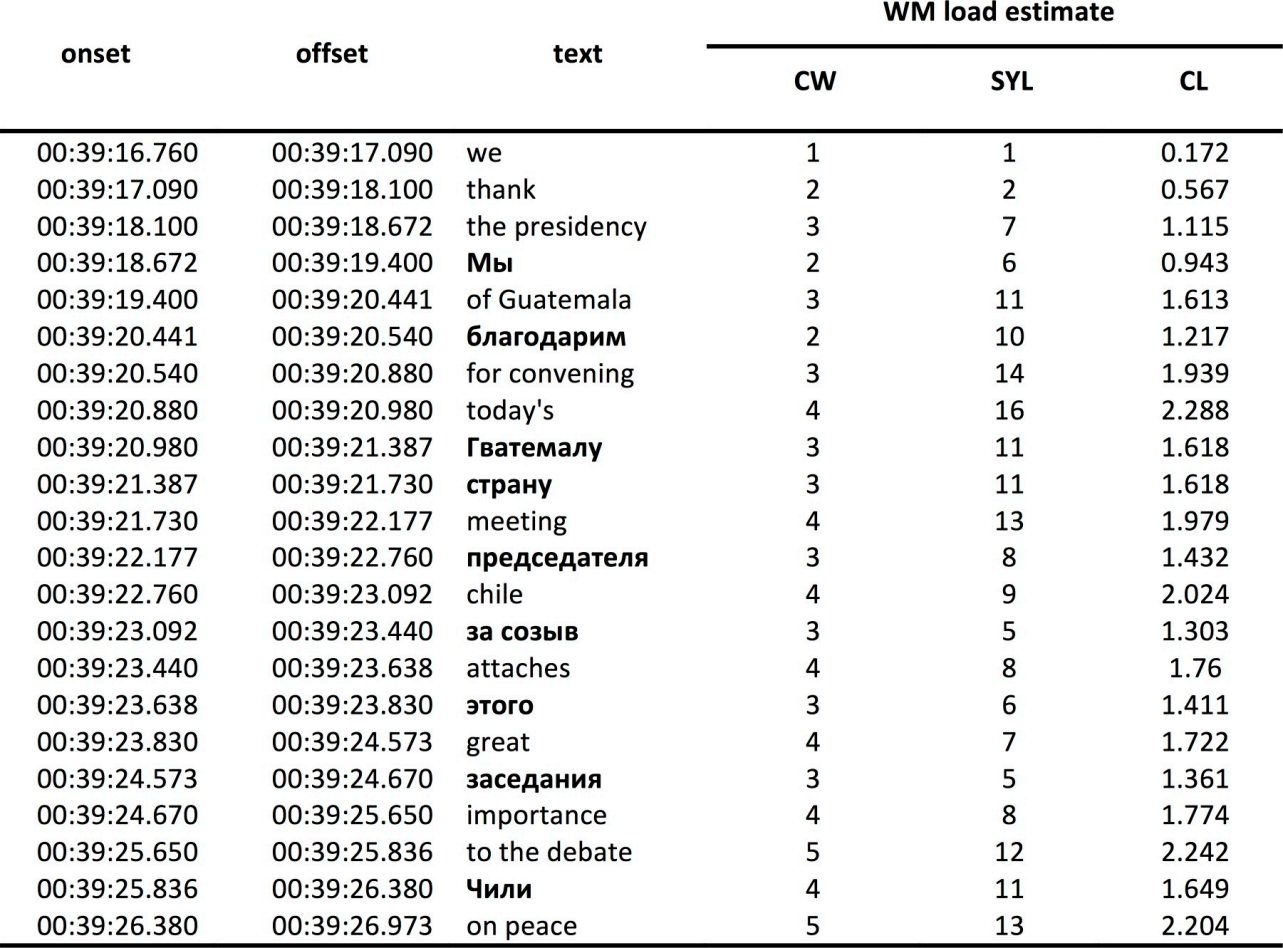
Illustration of a time-coded transcript. WM load is estimated based on the number of content words (CW), all the words (both function and content) weighted by their min-max normalized log-transformed frequency in the respective language corpus (CL), and content words weighted by their respective syllabic length (SYL).

After the experiments, we made transcripts of translations produced in relation to the original source speech. These were manually time-stamped and reformatted to allow us to calculate the different WM load estimates as described above. This work was partially automated using several custom functions in VBA running under Microsoft Excel. Fig 2 illustrates the resulting data.

### EEG data acquisition and pre-processing

The electroencephalogram (EEG) was continuously recorded using the ActiCHamp recording system and BrainVision PyCorder software (Brain Products GmbH) at the sampling rate of 2000 Hz from 32 scalp sites with active electrodes seated in an elastic cap.

EEG preprocessing and analysis were carried out using the EEGlab toolbox [55] for MATLAB (The Mathworks, Natick). Raw datasets were downsampled offline to 250 Hz, converted to a linked-mastoids reference (TP9 and TP10) and bandpass-filtered from 0.1 to 30 Hz (6 db/oct) using a zero-phase FIR filter. At the next step we ran independent component analysis (ICA) using the *binica* algorithm in EEGlab to remove the two most dominant independent components corresponding to oculomotor artifacts. Then, to address the issue of muscle noise produced by constant articulation in the experiment, we applied the artifact subspace reconstruction (ASR) algorithm [56]. The burst parameter was set to 4 standard deviations for mild cleaning of continuous EEG data.

To ensure the possibility of averaging the data epochs within specific WM load (*low*, *medium* or *high)*, we changed event codes in the EEG dataset to reflect WM load at the probe onset latency estimated with the three different methods described above.

The continuous EEG was then chunked into epochs of 500 ms post-stimulus (probe) onset including a 100 ms pre-stimulus baseline, yielding about 5840 epochs per subject. The epochs were screened for any residual artifacts that may have survived the ASR- and ICA-based cleaning stage. We then averaged the epochs within direction of interpretation and WM load estimated at time zero, i.e. the moment of probe onset, using each of the three methods.

### Data analysis

After EEG data pre-processing, we performed several statistical tests of our hypotheses. First, we wished to check for possible main effects (and interaction) of WM load and interpretation direction on the average ERP amplitude and peak latency in the N1 and P1 range. Following the recommendations of Handy [57], we used narrow time windows surrounding the observed peaks in the grand average ERP waveform, specifically 40–80 ms (for P1) and 120–160 ms (for N1) post stimulus onset, from which window-mean amplitudes were extracted. We used a 4-way repeated measures analysis of variance (ANOVA) with Direction (L1→L2, L2→L1), WM Load (Low, Medium, High), Anteriority (Front, Center, Back) and Laterality (Left, Middle, Right) as factors. Unless stated otherwise, all the statistical tests were done in R (R Development Core Team 2016) using the *ez* library.

## Results

### WM load estimates from behavioral data

**Fig 3:**
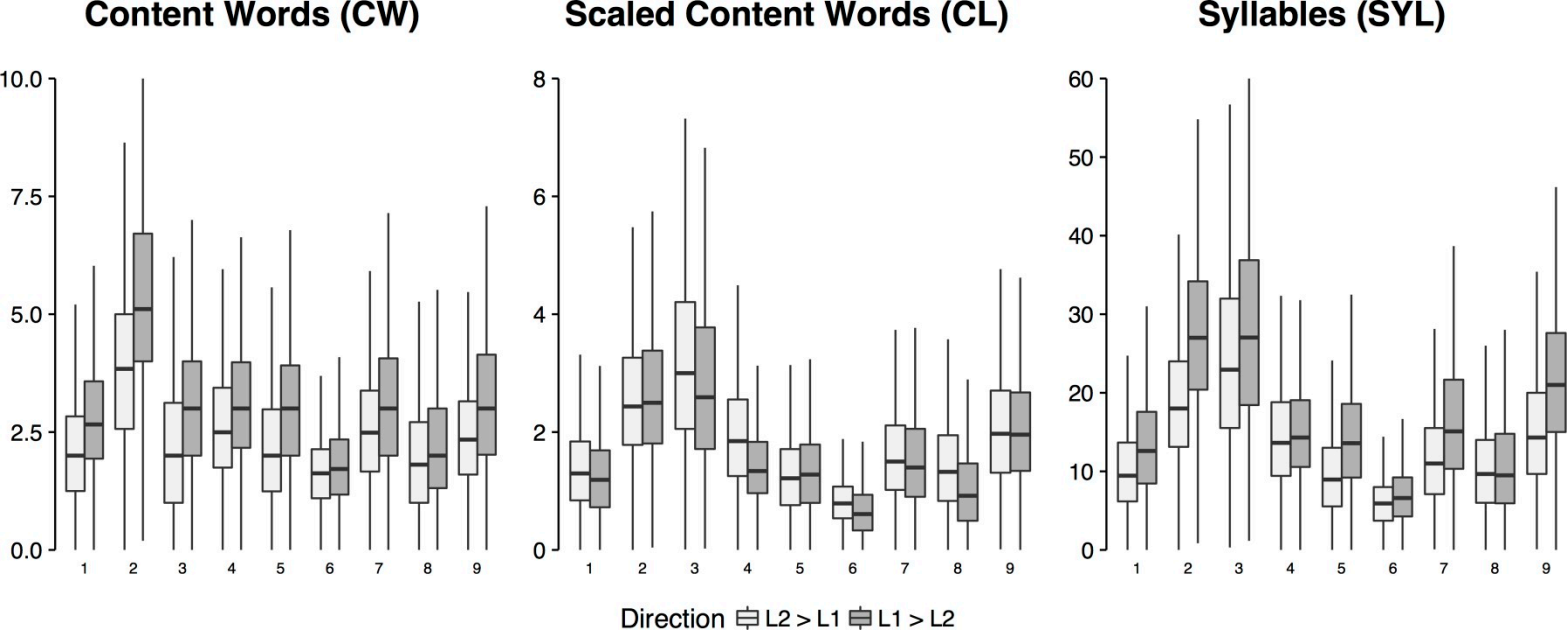
Distributions of WM load estimates by subject and direction of translation calculated using the three different methods.

Fig 3 shows the distributions of WM loads by participant and direction of interpretation. We first consider the distributions of WM load estimated by the most straightforward CW and SYL methods described above. All the participants’ median WM loads estimated with the CW and SYL methods were smaller in the L2→L1 direction than in the opposite direction. Since all the WM load distributions were clearly right-skewed, a non-parametric test was in order. A Wilcoxon signed rank test showed this difference to be significant: *V* = 45, *p =* 0.003906. However, the difference between WM loads calculated using the other method (CL) did not reach full statistical significance: *V* = 6, *p* = 0.05469.

### EEG results

To obtain an equal number of observations within each cell of our fully crossed experimental design, the boundaries between the high, medium and low WM load conditions should have been set at the 33^rd^ and 66^th^ quantiles of each subject’s WM load distributions (both for English-Russian and Russian-English). However, as can be inferred from Fig 3, there is a certain ‘comfort zone’ that interpreters keep to (or are forced to keep by the very conditions of the interpretation task) most of the time. Therefore, splitting the epochs into three equal WM load groups may not capture a potential effect on the neural activity due to the associated WM load. With that in mind, we labeled an epoch as *low* if the WM load at the probe onset was below the 10th quantile, *medium* if the WM load fell between the 10^th^ and 90^th^ quantile, and *high* if it exceeded the 90^th^ quantile in the corresponding WM load distribution. Although such a choice may seem arbitrary, it was a tradeoff between maximizing the effect size and sacrificing statistical power due to inflated error in the *low* and *high* WM condition. Mindful of large between-subject and between-language variance in the WM load distributions, we calculated the boundary quantiles for each participant and direction of translation individually obtaining 18 pairs (9 participants x 2 source language) of subject- and direction-specific condition boundaries.

**Fig 4:**
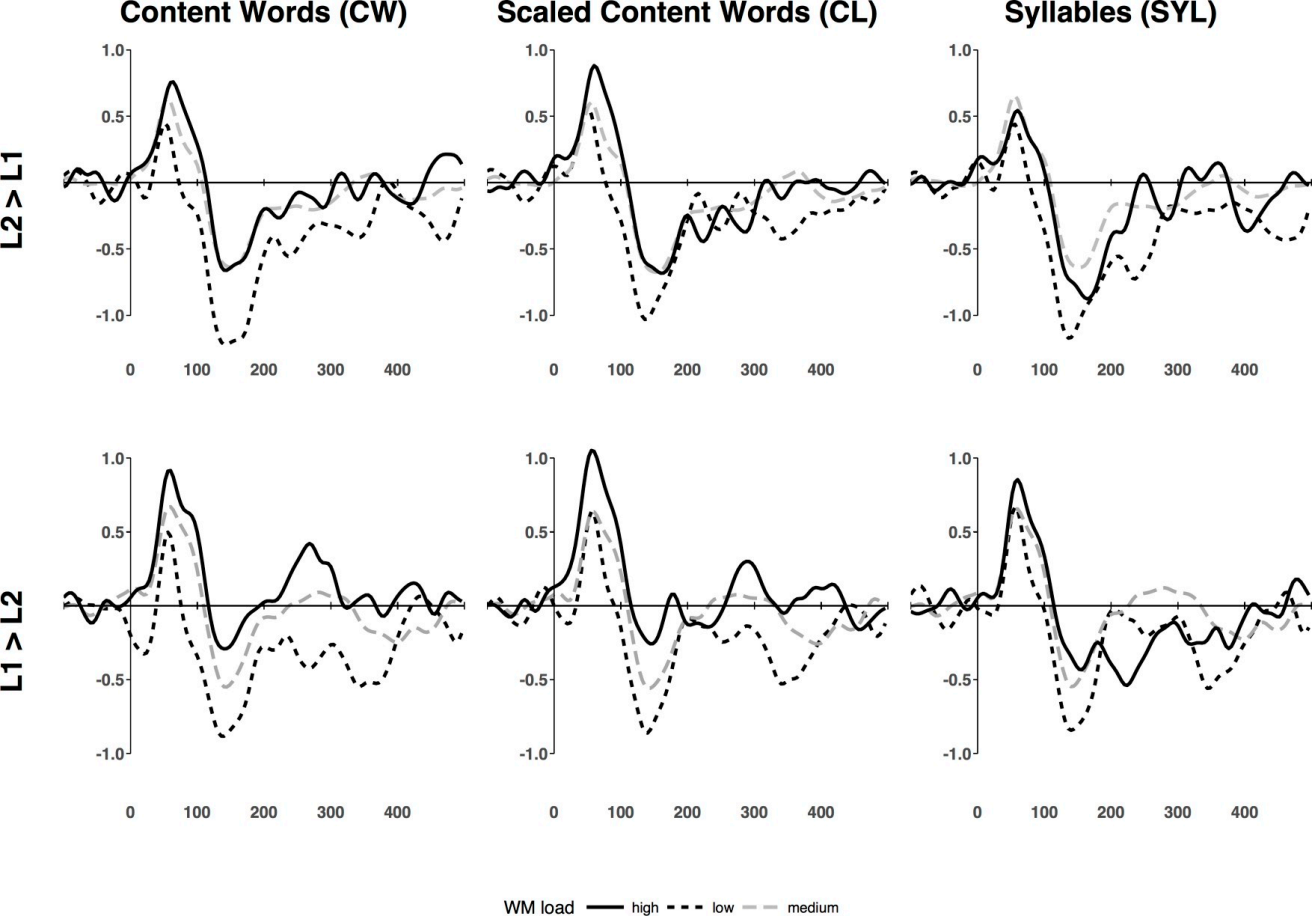
Grand average ERPs for L1 (Russian) → L2 (English) and L2 (English) → L1 (Russian) direction at Cz. The ERPs are plotted for the three different methods of estimating WM load. Y-axes display microvolts and X-axes milliseconds.

**Fig 5:**
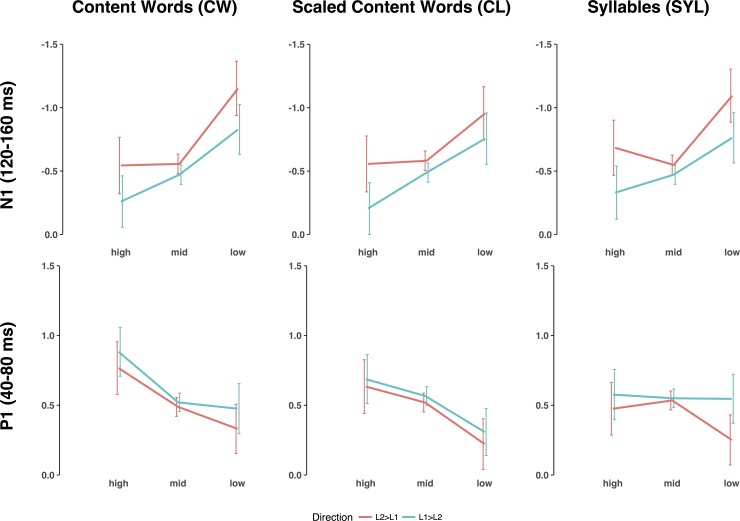
Grand average P1 and N1 amplitudes at Cz as a function of WM load. Vertical bars represent standard errors of the mean. None of the interactions reached significance. The main effect of WM load was significant for CW and CL (marked with an asterisk), see text for details.

**Fig 6:**
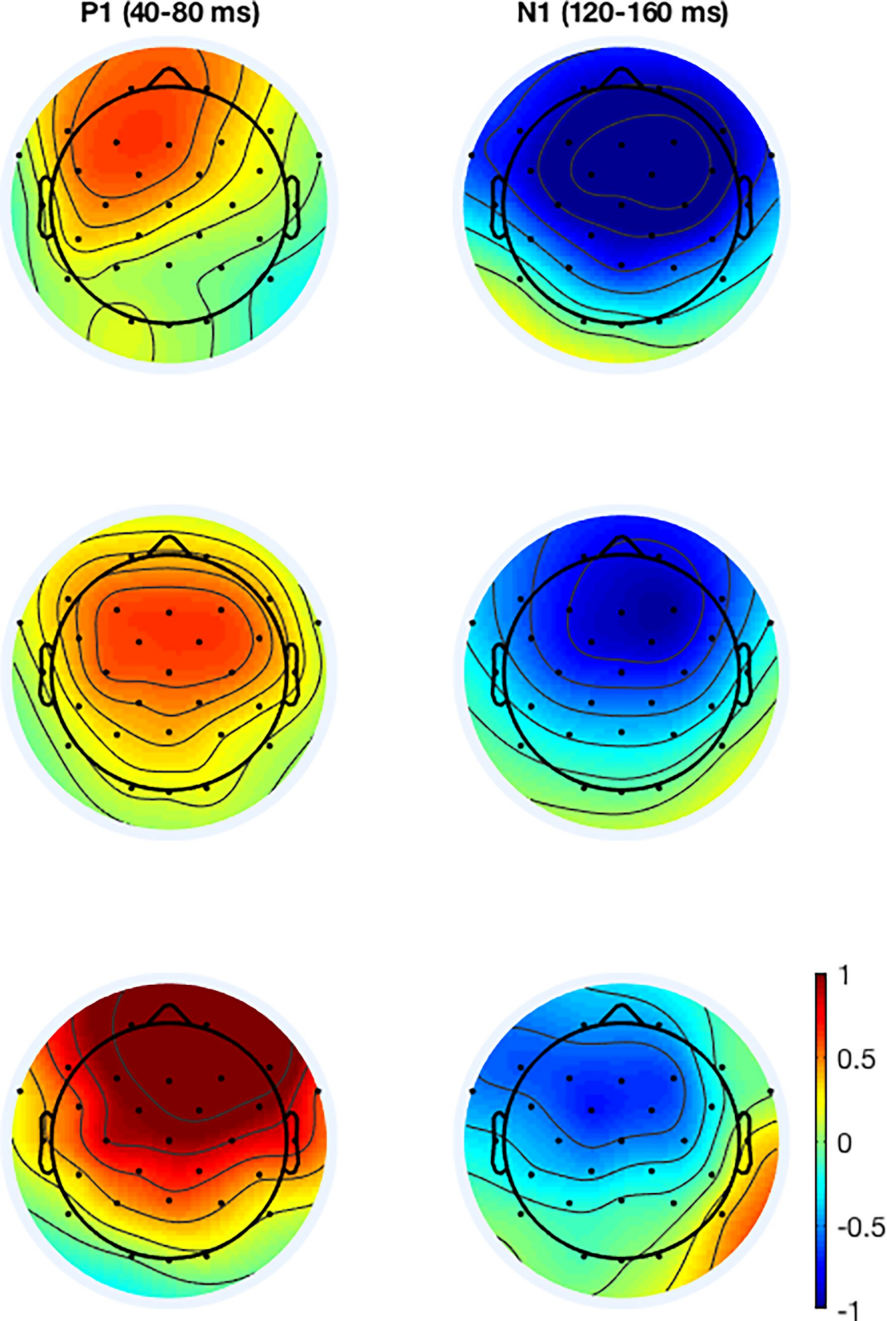
Grand average ERP topographical distributions. The topographical distributions are shown for low (A), medium (B) and high (C) WM load. WM load estimation method: CL.

Figs 4 and 5 show grand average ERPs at the Cz channel as a function of WM load. Fig 6 shows the topographical distributions computed on grand average ERP data. It is clearly seen that the P1 peak (~60 ms post stimulus onset) was topographically centered around frontal midline electrodes (Fz and Cz), while the N1 peak is slightly offset to the right. Accordingly, P1 and N1 amplitude and latency analyses were based on the following subset of electrodes: Fp1, Fz, F3, F7, FC5, FC1, C3, CP5, CP1, Pz, P3, P4, CP6, CP2, Cz, C4, FC6, FC2, F4, F8, Fp2.

#### P1 amplitude analyses

Regardless of the WM load estimation method used, we found neither a main effect of interpretation direction (CL [*F*(1, 8) = 0.20, *p* = 0.663, *η*^2^_p_ = 0.02]; CW [*F*(1, 8) = 0.34, *p* = 0.577, *η*^2^_p_ = 0.04]; SYL [*F*(1, 8) = 0.52, *p* = 0.491, *η*^2^_p_ = 0.06]), nor WM Load × Direction interaction (CL [*F*(1.07, 8.56) = 0.19, *p* = 0.690, *η*^2^_p_ = 0.02]; CW [*F*(2, 16) = 0.64, *p* = 0.542, *η*^2^_p_ = 0.07]; or SYL [*F*(1.13, 9.03) = 0.54, *p* = 0.502, *η*^2^_p_ = 0.06]). The main effect of WM load reached significance for the two methods based on the number of content words in the source-target lag, with smaller P1 amplitudes under lower WM load (CL [*F*(1.23, 9.86) = 5.02, *p* = 0.044, *η*^2^_p_ = 0.39]; CW [*F*(2, 16) = 3.94, *p* = 0.041, *η*^2^_p_ = 0.33], but not the one based on syllables (SYL [*F*(2, 16) = 0.12, *p* = 0.887, *η*^2^_p_ *=* 0.1]). Although the effect of WM load was stronger over the frontal sites, the Anteriority × Load interaction reached significance only for the CL method [*F*(4, 32) = 7.64, *p* < 0.001, *η*^2^_p_ = 0.49], but not for the other methods (CW [*F*(1.41, 11.32) = 1.48, *p* = 0.261, *η*^2^_p_ = 0.16; SYL [*F*(1.57, 12.58) = 0.17, *p* = 0.796, *η*^2^_p_ = 0.02]).

#### N1 amplitude analyses

Similarly to the P1 range, we found neither main effect of interpretation direction (CL [*F*(1, 8) = 0.03, *p* = 0.859, *η*^2^_p_ < 0.01]; CW [*F*(1, 8) = 0.53, *p* = 0.486, *η*^2^_p_ = 0.06]; or SYL [*F*(1, 8) = 1.11, *p* = 0.323, *η*^2^_p_ = 0.12]), nor WM Load × Direction interaction (CL [*F*(2, 16) = 0.03, *p* = 0.967, *η*^2^_p_ < 0.01]; CW [*F*(2,16) = 0.07, *p* = 0.929, *η*^2^_p_ < 0.01]; or SYL [*F*(1.18, 9.42) = 0.20, *p* = 0.707, *η*^2^_p_ = 0.02]). However, the main effect of WM load was consistently significant for all the WM load estimation methods, with larger N1 amplitudes under lower WM load (CL [*F*(2, 16) = 7.99, *p* = 0.04, *η*^2^_p_ = 0.50]; CW [*F*(2, 16) = 11.31, *p* < 0.001, *η*^2^_p_ = 0.59]; and SYL [*F*(2, 16) = 8.94, *p* = 0.002, *η*^2^_p_ = 0.53]). As in the P1 range, the effect of WM load was more pronounced over the frontal sites. However, the Anteriority × Load interaction did not reach significance (CL [*F*(1.65, 13.20) = 2.49, *p* = 0.127, *η*^2^_p_ = 0.24]; CW [*F*(2, 15.98) = 2.48, *p* = 0.115, *η*^2^_p_ = 0.24]; SYL [*F*(1.64, 13.12) = 0.94, *p* = 0.397, *η*^2^_p_ = 0.11]).

Considering the modest sample size, we performed an additional analysis of N1 and P1 amplitudes using the jackknife method as described by Miller, Ulrich and Schwartz [58]. Specifically, we took leave-one-out resamples from the data such that each of them included all but one participant’s data, and ran the same statistical tests on each resample in order to ensure that the results were not driven by a few outliers or other spurious effects; this has fully confirmed our main analysis.

#### P1 and N1 latency analyses

The ANOVA on P1 peak latency data showed a significant main effect of WM load for CL, *F*(2, 16) = 4.90, *p* = 0.022, *η*^2^_p_ = 0.38. Specifically, P1 latency was smaller for low WM load (*M* = 59.66, *SE* = 0.043) than for high WM load (*M* = 60.31, *SE* = 0.043). No such effect was significant for CW or SYL. No other significant main effects or interactions were observed.

No significant main effects or interactions were observed from the ANOVA of N1 peak latency data for any of the WM load estimation methods.

## Discussion

Although several authors have investigated functional and structural plasticity induced by SI training and practice (e.g. [59,60]), we are not aware of any attempts to validate models of SI using neuroimaging methods, and in the present study we tried to address this gap. Specifically we aimed to electrophysiologically test the Efforts Model of SI [13,15,37] which predicts that WM overload during simultaneous interpretation should decrease the amount of attention available to support the ‘listening effort’ (that the Efforts Model defines as non-automatic operations involved in auditory perception and analysis of auditory stimuli) and therefore degrade the processing of the source message. Although current WM load can be conveniently estimated if we assume it to be proportional to the size of lag between the source and the target (in the number of content words), getting a precise behavioral measure of attention during SI would have been problematic. However, as was previously demonstrated in non-interpreters, the amplitude of early ERP components time-locked to task-irrelevant probe stimuli appears more negative when the target audio is attended [17], and therefore could serve as an electrophysiological index of attention. Based on that and the prediction of the Efforts Model, we expected a larger negativity in the P1 and N1 range at small WM loads suggesting enhanced processing of the current portion of the source message.

Consistent with our hypothesis, we found that the amplitude of the P1 and N1 component elicited by the task-irrelevant pure tone probes was more negative at lower WM loads. Conversely, the P1 and N1 amplitude was less negative at higher WM loads suggesting that the brain temporarily attenuates or suspends the processing of auditory stimuli to more efficiently process and manipulate WM backlog, reduce the lag and cognitive load. The question whether this is controlled consciously (i.e. is a choice based on strategic judgment), or automatically (i.e. is a skill acquired though training and used unconsciously) has been treated in previous research (e.g. [61–63]) and still remains underexplored. A correlational study incorporating behavioral performance measures (such as WM span) on a larger sample of interpreters with different levels of experience may in the future help answer the question with more certainty.

A visual inspection of the ERP waveforms revealed that the effect of WM load in the P1/N1 range may not have been due only to enhanced negativity, but also to decreased latency of the P1 and N1 peaks for low WM load. Fehér, Folyi & Horváth [64] reported a shorter N1 latency for attended stimuli, which reinforces the case for attention acting as a latent variable mediating the effect of WM load on the early ERP components. Although our results did suggest greater P1 and N1 latencies at larger WM load, the effect was statistically significant only for the P1 range.

As part of our second research question, we wished to establish if the subjectively greater difficulty of SI from L2 into L1 reported by the respondents of a separate survey, was due to direction-specific WM load management differences and, if any, to find their electrophysiological correlates. Although the behavioral data showed a pattern of smaller median WM loads in the L2→L1 direction, which seems to be consistent with the predictions of the Efforts Model, we found no significant main effect of direction on the ERPs (P1/N1) or interaction between direction and WM load. One possible explanation could be that although on average keeping a certain number of L2 words in WM does require more effort than the same number of L1 words, the overall level of attention to the source message stays the same regardless of the language of input. In other words, in the L2→L1 direction interpreters’ attention to the source message is not inferior compared to L1→L2 direction, because the increased effort involved in processing L2 input in WM is compensated by maintaining WM load at a level lower than in L1→L2 direction.

The finding of significantly different median WM loads for the two interpretation directions may be due to several reasons. First, because L2 words on average are less ‘familiar’ than L1 words, they have weaker mental representations than the corresponding L1 words. As a result, L2 words are known to take more time to react to in lexical decision tasks [50]. For our participants (Russian L1, English L2 speakers) this indicates that the perception and analysis of L2 speech is more difficult than L1. Moreover, this difficulty is not offset by the relative ease of producing a meaningful target message in the mother tongue, which is evidenced by shorter lexical retrieval times for L1 words (e.g. [65]). Second, WM is less efficient in keeping and manipulating L2 words than L1 words [38]. Perhaps, when working from L2 into L1, interpreters conserve processing capacity by reducing WM load, while in the opposite direction they can afford to keep in WM more L1 than L2 content words. Third, interpreting from Russian into English—again, all else being equal—appears less of a challenge because Russian words are on average syllabically longer than in English [66]. Thus, a source message in Russian is informationally more sparse and easier to process than a comparable English message delivered at the same rate measured in syllables per unit time. Therefore, in English-Russian interpretation, the target Russian discourse is syllabically longer and therefore should cause more interference due to acoustic overlap with the source message. In other words, if we assume word-for-word rendering, the interpreter has less time to ‘unpack’ the English message: English words are shorter than Russian requiring more time to complete the articulation of the corresponding Russian translation. Not only does this create greater and potentially disrupting acoustic interference with the process of listening to the English source, it is possible that attention is engaged longer in the self-monitoring stage of the interpretation cycle. However, at least one study [67] based on seven languages—unfortunately, not including Russian—found that in spoken communication the average amount of information transferred per unit time is subject to substantial, although statistically insignificant, cross-language variation. It is possible that the obvious difference in WM loads as a function of source language is due to different information densities of the languages used here.

Although it is impossible to directly generalize our results to situations with any other source-target language pair, our data suggest that the relative share of attentional resources allocated to the ‘listening effort’ during SI does not depend on the language of input. We can at least argue that the main source of subjective difficulty in the English-Russian interpreting does not lie in input language-specific distribution of attention.

While enhanced negativity in the P1/N1 range under low WM load during SI fits well with the Efforts Model and task-engagement/distraction trade-off model [68,69], there is room for another interpretation. It is possible that when WM load increases, the task-irrelevant probes are processed less, while the task-relevant source message still receives the same amount of attention as it does under low WM load. This could be an interesting subject of a follow-up investigation.

Although the number of participants in the present study was relatively small, by limiting our selection to professionally trained highly-skilled individuals for whom interpreting is a main occupation rather than a side activity, we believe that we could focus on a population that can in fact be characterized as a core group of conference interpreters, rather than mere L2 speakers tasked with an interpreting job. Notably, we were able to verify the results using the more elaborate jacknife analytical statistics tools which fully confirmed our original analysis; that said, future studies will benefit from attempting to collect larger samples of professional interpreters, albeit this is not a trivial task.

On a practical note, our results justify a recommendation to keep WM load within reasonable limits during SI. The question about how this can be achieved is very relevant. Several studies [7,70,71] have shown that simultaneous interpreters use a range of strategies to manage their processing load (e.g. omitting redundancies in the source speech). None of them, however, have attempted to identify the neural states that determine—or at least bias—the choice of a particular strategy.

Further neuroimaging studies are needed to validate and/or refine theoretical models of SI and will help to guide interpreters, SI instructors and the interpretation industry towards better interpretation practices, more efficient curricula and higher standards of service.

## Limitations and future directions

One major limitation of the study is the narrow group of participants that only included L1 Russian/L2 English participants. While our results do lend support to the Efforts Model of simultaneous interpreting, one should be cautious in directly generalizing its results to any other subpopulations of conference interpreters whose L1 and L2 are other than Russian or English. Every language presents a unique set of difficulties at multiple levels, including syntactic, lexical and phonetic, which potentially influence interpreting strategies including the lag and overall working memory load. For example, when working from German, interpreters are forced to increase their décalage [12] waiting for the predicate that often appears at the very end of the clause, which means a larger WM load overall. An especially interesting direction for future research is to conduct similar experiment on L1 English speakers translating the same speeches to see if the pattern of results would be consistent with the predictions of the Efforts Model.

Another limitation of the study relates to possible performance differences that may exist across the different interpreters. To address this limitation, future research could implement a more advanced design incorporating behavioral performance measures (e.g. working memory span and speed of lexical access) into the overall data analysis and interpretation of results. It would be interesting to see if these performance measures are correlated with average WM load during the performance of an SI task.

Finally, it has to be acknowledged that our study did not analyze the role of the speaking effort–the third essential component of the Efforts Model–not least because of the severe difficulties of overcoming articulation artifacts in EEG analysis. Future EEG studies may provide more valuable insights into the microdynamics of cognitive processes during simultaneous interpreting and help advance the state of the art in conference interpreter training.

## Supporting information

S1 Appendix Parameters of speeches used in the interpreting task. (DOCX)

S2 Appendix The order in which the speeches were presented. (DOCX)
