# Supplementary material for: Testing the efforts model of simultaneous interpreting: An ERP study
Source: PLoS One. 2018 Oct 24;13(10):e0206129. doi: 10.1371/journal.pone.0206129 (PMC6200263; doi:10.1371/journal.pone.0206129)
Supplement: S1 Appendix — (DOCX) [file pone.0206129.s001.docx]

| **Code** | **Country** | **Original Speakers' Mother Tongue** | **Interpreting Task** | **Words in the recording** | **Edited Duration, s** | **WPM Rate** |
| --- | --- | --- | --- | --- | --- | --- |
| a | France | French | Ru 🡪 En | 1000 | **571** | 105 |
| b | Morocco | French | En 🡪 Ru | 769 | **439** | 105 |
| c | Honduras | Spanish | Ru 🡪 En | 606 | **346** | 105 |
| d | Colombia | Spanish | En 🡪 Ru | 1081 | **618** | 105 |
| e | Peru | Spanish | Ru 🡪 En | 654 | **374** | 105 |
| f | Costa Rica | Spanish | En 🡪 Ru | 608 | **347** | 105 |
| g | Uruguay | Spanish | Ru 🡪 En | 502 | **287** | 105 |
| h | Chile | Spanish | En 🡪 Ru | 520 | **297** | 105 |
