# Supplementary material for: Testing the efforts model of simultaneous interpreting: An ERP study
Source: PLoS One. 2018 Oct 24;13(10):e0206129. doi: 10.1371/journal.pone.0206129 (PMC6200263; doi:10.1371/journal.pone.0206129)
Supplement: S2 Appendix — (DOCX) [file pone.0206129.s002.docx]

|  | *block 1* | *block 2* | *block 3* | *block 4* | *block 5* | *block 6* | *block 7* | *block 8* |
| --- | --- | --- | --- | --- | --- | --- | --- | --- |
| *Participant 1* | a | b | c | d | e | f | g | h |
| *Participant 2* | b | c | d | e | f | g | h | a |
| *Participant 3* | c | d | e | f | g | h | a | b |
| *Participant 4* | d | e | f | g | h | a | b | c |
| *Participant 5* | e | f | g | h | a | b | c | d |
| *Participant 6* | f | g | h | a | b | c | d | e |
| *Participant 7* | g | h | a | b | c | d | e | f |
| *Participant 8* | h | a | b | c | d | e | f | g |
| *Participant 9* | a | b | c | c | e | f | g | h |
